# Hospitalization Costs of COVID-19 Cases and Their Associated Factors in Guangdong, China: A Cross-Sectional Study

**DOI:** 10.3389/fmed.2021.655231

**Published:** 2021-06-11

**Authors:** Moran Dong, Zuyao Yang, Yingyao Chen, Jiufeng Sun, Wenjun Ma, Shouzhen Cheng, Xiaoli Sun, Jianpeng Xiao, Guanhao He, Jianxiong Hu, Jiaqi Wang, Guimin Chen, He Zhou, Lixia Yuan, Jiali Li, Xuan Li, Hui Xu, Ruijie Wang, Dengzhou Chen, Ming Fang, Tao Liu

**Affiliations:** ^1^Guangdong Provincial Institute of Public Health, Guangdong Provincial Center for Disease Control and Prevention, Guangzhou, China; ^2^School of Public Health, Southern Medical University, Guangzhou, China; ^3^JC School of Public Health and Primary Care, The Chinese University of Hong Kong, Hong Kong, China; ^4^School of Public Health, Fudan University, Shanghai, China; ^5^Nursing Department, The First Affiliated Hospital, Sun Yat-sen University, Guangzhou, China; ^6^Gynecology Department, Guangdong Women and Children Hospital, Guangzhou, China; ^7^School of Exercise Science and Health, Guangxi College of Physical Education, Nanning, China; ^8^Department of Intensive Care Medicine, Guangdong Provincial People's Hospital, Guangdong Academy of Medical Sciences, Guangzhou, China; ^9^Department of Intensive Care Medicine, Guangdong Provincial People's Hospital-Nanhai Hospital, Foshan, China; ^10^School of Medicine, South China University of Technology, Guangzhou, China; ^11^School of Medicine, Jinan University, Guangzhou, China

**Keywords:** COVID-19, hospitalization costs, associated factors, traditional Chinese medicine therapy, China

## Abstract

**Background:** The ongoing COVID-19 pandemic has brought significant challenges to health system and consumed a lot of health resources. However, evidence on the hospitalization costs and their associated factors in COVID-19 cases is scarce.

**Objectives:** To describe the total and components of hospitalization costs of COVID-19 cases, and investigate the associated factors of costs.

**Methods:** We included 876 confirmed COVID-19 cases admitted to 33 designated hospitals from January 15th to April 27th, 2020 in Guangdong, China, and collected their demographic and clinical information. A multiple linear regression model was performed to estimate the associations of hospitalization costs with potential associated factors.

**Results:** The median of total hospitalization costs of COVID-19 cases was $2,869.4 (IQR: $3,916.8). We found higher total costs in male (% difference: 29.7, 95% CI: 15.5, 45.6) than in female cases, in older cases than in younger ones, in severe cases (% difference: 344.8, 95% CI: 222.5, 513.6) than in mild ones, in cases with clinical aggravation than those without, in cases with clinical symptoms (% difference: 47.7, 95% CI: 26.2, 72.9) than those without, and in cases with comorbidities (% difference: 21.1%, 21.1, 95% CI: 4.4, 40.6) than those without. We also found lower non-pharmacologic therapy costs in cases treated with traditional Chinese medicine (TCM) therapy (% difference: −47.4, 95% CI: −64.5 to −22.0) than cases without.

**Conclusion:** The hospitalization costs of COVID-19 cases in Guangdong were comparable to the national level. Factors associated with higher hospitalization costs included sex, older age, clinical severity and aggravation, clinical symptoms and comorbidities at admission. TCM therapy was found to be associated with lower costs for some non-pharmacologic therapies.

## Introduction

The ongoing COVID-19 pandemic, an emerging infectious disease caused by the novel severe acute respiratory syndrome coronavirus 2 (SARS-CoV-2), has now affected more than 210 countries, areas or territories worldwide. As of October 26th, 2020, more than 42 million confirmed cases and more than 1.1 million deaths have been reported (1). In order to “flatten the epidemic curve,” the global community has enforced border shutdowns, travel restrictions and quarantine, which has severely affected global socio-economic development (2). It was estimated that the current outbreak of COVID-19 leads to at least 1 trillion U.S. dollars ($1 trillion) loss to world's economy during year 2020, which is even worse than the 2008 Great Financial Crisis (3). In addition, the COVID-19 pandemic brought significant challenges to health system and consumed a lot of health resources. The large number of COVID-19 cases have occupied most heath care resources and caused many health care workers infected in some countries.

Understanding the hospitalization costs of COVID-19 cases and their associated factors has important implications for estimating the direct costs of COVID-19, and for clinical doctors to develop treatment strategies. However, few studies have reported such information in China where the COVID-19 epidemic was firstly reported (4, 5). A Chinese national report showed that the mean hospitalization costs of confirmed COVID-19 cases were $3,083.0, and the mean costs of severe cases was more than $21,500.0 (6). In Li et al. investigation conducted in 105 COVID-19 cases in Shenzhen, China, they found that the mean hospitalization costs were $1,762.0, and that the hospitalization costs were associated with age and duration of hospitalization (5). In addition, one report in the United States of America estimated that a single symptomatic COVID-19 infection would cost a median of $3,045 in direct medical costs incurred, and that patients' age, ICU admission and hospitalization would affect the costs (7). Some of these studies did not analyze the components of hospitalization costs, and did not account for the impacts of other factors such as presence of symptoms, comorbidities at admission, clinical aggravation during hospitalization, and strategies of therapy. Therefore, more investigations are needed.

As SARS-CoV-2 is a novel virus, there is no specific effective treatment particularly in the early stage of pandemic. Based on past experiences in the treatment of infectious diseases in China, Traditional Chinese Medicine (TCM), including herbal formulas, was widely used to treat COVID-19 cases (8). It was reported that early intervention with TCM in mild cases can effectively prevent them from progressing to severe conditions (9). Hence, it is plausible that the usage of TCM may reduce hospitalization costs of COVID-19 cases. However, few (if any) investigated the impacts of TCM on hospitalization costs.

## Methods

### Study Setting

Guangdong is a province with a large population size located in southern China, which is a place early affected by COVID-19. The first confirmed case was reported on January 15th, 2020, and a total of 1,819 confirmed cases were reported as of September 28th, 2020. We retrospectively selected admitted confirmed COVID-19 cases from 33 designated hospitals from January 15th, 2020 to April 27th, 2020 in Guangdong, China. These hospitals were designated by governments to receive and treat confirmed COVID-19 cases. There is at least one designated hospital in each city in Guangdong Province, China. Once a COVID-19 case was confirmed in a general hospital according to the Diagnosis and Treatment scheme of COVID-19 (10, 11), he/she would be immediately admitted to a near designated hospital. There were few cases after May, 2020 (Out of the total 1,819 confirmed cases up to September, 2020, only 231 cases were confirmed after May), and most of them were imported cases. Some important information of hospitalization costs in those imported cases was not available. Therefore, we selected days from January 15th, 2020 to April 27th, 2020 as our study period. Moreover, those confirmed cases (712 cases) without information of key variables such as hospitalization costs, drug usage and non-pharmacologic therapy were excluded.

### Data Collection

Information of all included cases were obtained from the Guangdong Provincial Office of COVID-19 Control and Prevention, which was set and designated by the government. Following the Law of the China on the Prevention and Treatment of Infection Diseases, and the Emergency Regulations Regarding Emergency Public Health Incidents, each confirmed COVID-19 case's information must be reported to the office, and was used for making and adjusting policies.

We collected the following information of each included case from medical record system and treatment system of all designated hospitals after they discharged from hospital: demographic characteristics (sex, age, and days of hospitalization), hospitalization costs, including total costs, drug usage costs (the cost of TCM and western medicine), examination costs (such as blood routine, urine routine, liver function, D-dimer, and B-ultrasound), non-pharmacologic therapy costs (the fee charged by the medical staff for the relevant operation, such as infusion fee, suture removal fee, atomization inhalation fee, physical therapy fee, and nursing fee) and others costs (the cost of materials, such as disposable infusion set, and disposable syringe) (Supplementary Table 1), symptoms at admission, comorbidities, severity, clinical aggravation during hospitalization, drug usage, and non-pharmacologic therapy information, TCM therapy, and general information of the designated hospital. All data used in this study were anonymous and without identifiable private information.

### Definitions of Key Covariates

The hospital level was divided into two categories: tertiary hospital (typically larger and comprehensive), and secondary hospital (often regional, relatively smaller) (12). Clinical severity at admission was divided into three categories according to the Diagnosis and Treatment Protocol for Novel Coronavirus Pneumonia released by the National Health Commission of the People's Republic of China: mild, moderate and severe (13). A case would be defined as aggravation by clinical doctors if his/her clinical situation becomes severer during hospitalization such as from mild to moderate. Symptoms and comorbidities at admission were also collected by clinical doctors.

### Statistical Analysis

For categorical variables, we calculated the percentages (%) of cases in each category. Because the distribution of hospitalization costs was positive skewedness distribution, we used the median and interquartile range (IQR) to describe the hospitalization costs of COVID-19 cases in each category. A multiple linear regression model was performed to evaluate the associations between hospitalization costs and potential associated factors including demographic characteristics, symptoms at admission, severity of cases at admission, clinical aggravation during hospitalization, comorbidities, drug usage, non-pharmacologic therapy, TCM therapy, and other factors. In the multiple linear regression model, three variables were adjusted for as potential confounders including sex, age, and hospital level at admission. Collinearity diagnosis was performed to test the potential collinearity among the three adjusted for confounders and independent variables, and only variables with variance expansion factor (VIF) <2 were included in multiple linear regression (14). The hospitalization costs were transformed by natural logarithm (ln), and the natural log-scaled partial coefficient of linear regression can be exponentiated to express the percentage changes [% difference and its 95% confidence interval (CI)] of the hospitalization costs (15).

In particular, we tested the differences in drug usage costs and non-pharmacological therapy costs between TCM group and non TCM group after adjustment for potential confounders (sex, age, clinical severity, and hospital level at admission) in the total cases and several subgroups, which was used to particularly examine the effects of TCM on hospitalization costs. All data analyses were conducted by R software (version 3.6.3, R Foundation for Statistical Computing).

### Ethics Approval Statement

This study was approved by the Ethics Committee of Guangdong Provincial Center for Disease Control and Prevention (No. W96-027E-2020004). The data analysis was carried out at a population level after data aggregation. We didn't contact any individual subjects.

## Results

### General Characteristics of COVID-19 Cases

A total of 876 COVID-19 cases were included for analysis in this study. Table 1 shows the general characteristics of all participants. Out of the total participants, 442 (50.5%) were males, 621 (70.9%) were aged 20–59 years, and 802 (91.6%) were admitted to tertiary hospitals. At the time of admission, 73 (8.3%), 760 (86.8%), and 43 (4.9%) cases were categorized to mild, moderate, and severe groups, respectively, 733 (83.7%) cases had clinical symptoms, and 222 (25.3%) cases had comorbidities at admission. During the hospitalization, 150 (17.1%) patients aggravated in which four (0.5%) cases died, 647 (73.9%) were hospitalized for more than 15 days, and 45 (5.9%) cases were treated with TCM therapy (Table 1).

**Table 1:** General characteristics of COVID-19 cases in Guangdong Province, China.

**Characteristics**	***N* (%)**
Sex	
Female	434 (49.5)
Male	442 (50.5)
Age (years)	
<19	79 (9.0)
20–29	124 (14.2)
30–39	203 (23.2)
40–49	129 (14.7)
50–59	165 (18.8)
60–69	137 (15.6)
≥70	39 (4.5)
Hospital level of admission	
Secondary hospital	74 (8.4)
Tertiary hospital	802 (91.6)
Clinical severity at admission	
Mild	73 (8.3)
Moderate	760 (86.8)
Severe	43 (4.9)
Antiviral drugs usage	
No	812 (92.7)
Yes	64 (7.3)
ECMO therapy^a^	
No	865 (98.7)
Yes	11 (1.3)
Clinical aggravation during hospitalization	
No	726 (82.9)
Yes	150 (17.1)
Death during hospitalization	
No	872 (99.5)
Yes	4 (0.5)
Comorbidity	
No	654 (74.7)
Yes	222 (25.3)
Clinical symptoms at admission	
No	143 (16.3)
Yes	733 (83.7)
Days of hospitalization	
0–14	227 (25.9)
15–21	289 (33.0)
22–28	163 (18.6)
>28	195 (22.3)
Unknown	2 (0.2)
TCM therapy^b^	
No	831 (94.9)
Yes	45 (5.1)
ICU therapy^c^	
No	827 (94.4)
Yes	49 (5.6)

a *ECMO, Extracorporeal membrane oxygenation*.

b *TCM, Traditional Chinese Medicine*.

c *ICU, Intensive care unit*.

**Figure 1:**
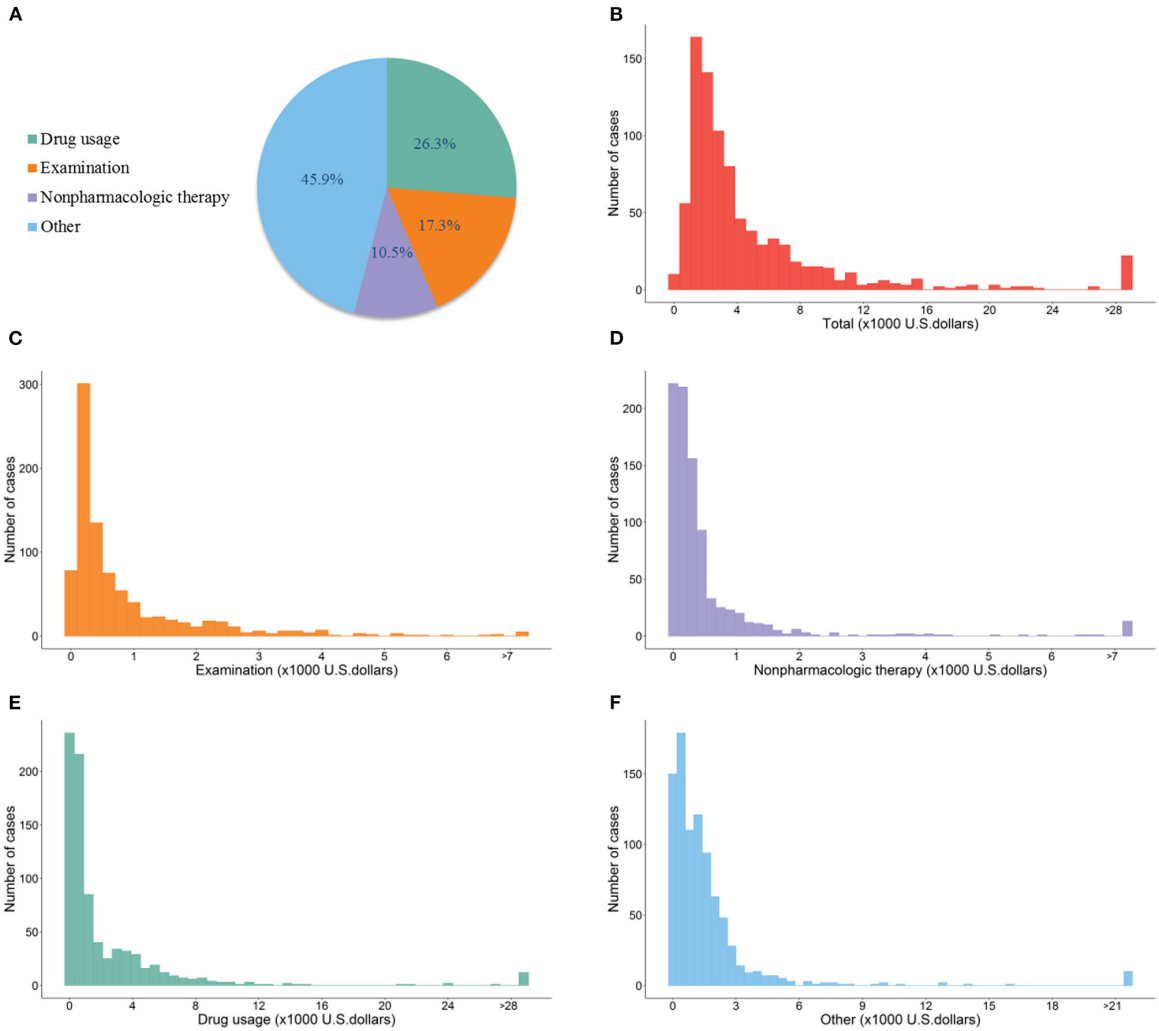
Distribution of hospitalization costs of COVID-19 cases in Guangdong Province, China. **(A)** (Pie graph) shows the components of the total hospitalization costs, including examination costs, non-pharmacologic therapy costs, drug usage costs, and other costs. **(B–F)** Show the frequency of total hospitalization costs, examination costs, non-pharmacologic therapy costs, drug usage costs, and other costs in all included COVID-19 cases in Guangdong Province, China. The costs were presented in U.S. dollars (×1,000). The bars in these panels represent the number of cases.

**Figure 2:**
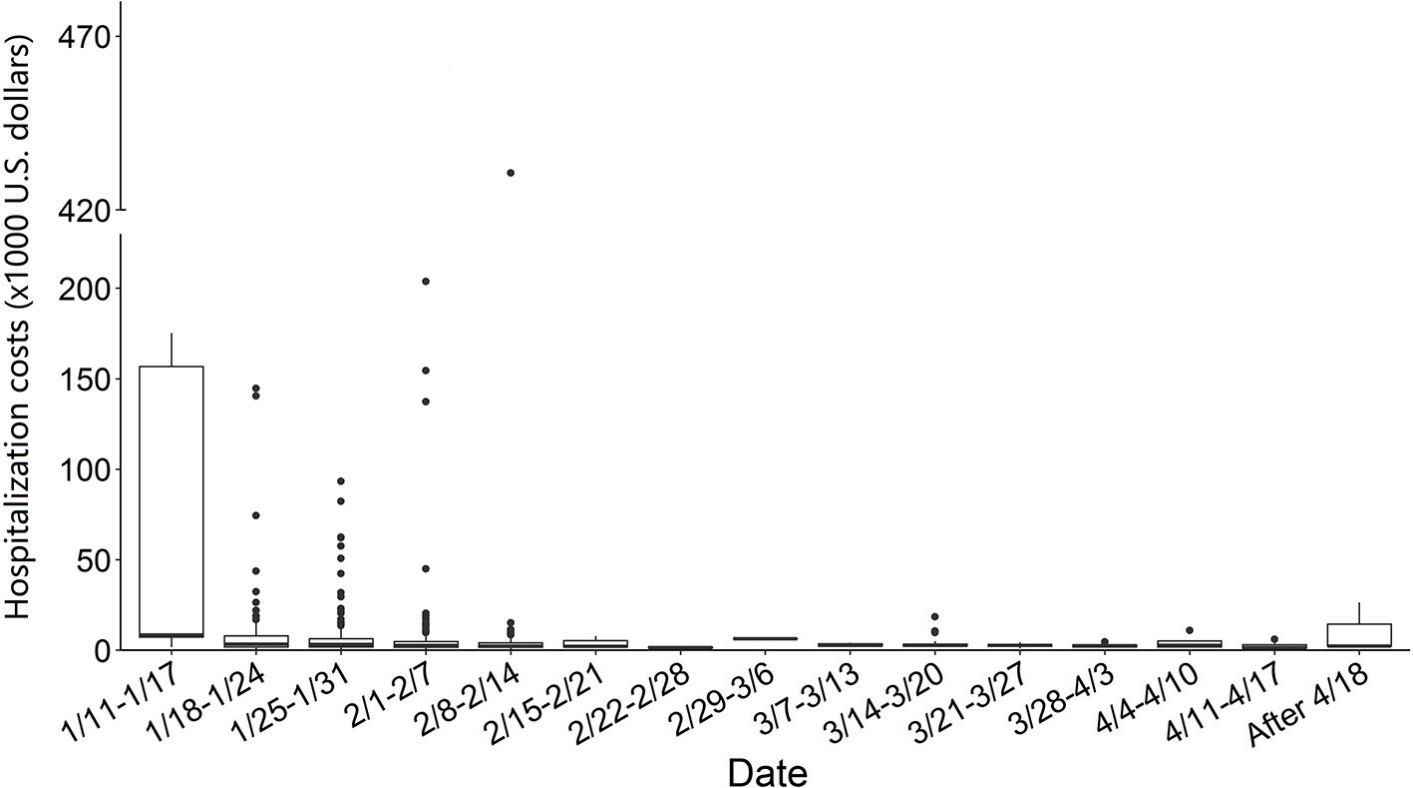
Weekly hospitalization costs distribution of COVID-19 cases from January 11th to April 27th in Guangdong Province, China. The central line represents the median of distribution, boxes span the 25th to the 75th centiles, and the error bars represent the minimum and maximum values after excluding those outliers which were marked by dots.

Figure 1 shows the distribution of total hospitalization costs and its components in the total cases. The median of total hospitalization costs in all cases was $2,869.4 IQR: $3,916.7 with the maximum of $0.4 million, and the minimum of $53.0. Out of the total cases, 94% had total hospitalization cost <$14,347.2. The total costs were consisted of drug usage (26.3%), examination (17.3%), non-pharmacologic therapy (10.5%), and other costs (45.9%). The median costs of drug usage, examination, non-pharmacologic therapy, and others were $631.3 (IQR: $2,539.5), $416.1 (IQR: $789.1), $258.3 (IQR: $401.7), and $1,047.3 (IQR: $1,477.8), respectively. Figure 2 demonstrates the weekly average hospitalization cost in all cases from January 11th, 2020 to April 27th, 2020. A general decreasing trend was found during the study period. Several cases with extremely high costs were observed before February 15th, after which the costs of most cases were <$14,347.2.

### Associated Factors of the Total Hospitalization Costs in COVID-19 Cases

Tables 2, 3 shows the factors that influence the hospitalization costs of COVID-19 cases. We found higher hospitalization costs in male cases (% difference: 29.7, 95% CI: 15.5, 45.6) than in female cases, in older cases than in younger cases (e.g., % difference: 323.0, 95% CI: 202.9, 490.7 for cases over 70 years compared with cases under 19 years), in cases admitted in the tertiary hospitals (% difference: 51.3, 95% CI: 22.8, 86.3) than in the secondary hospitals, and in cases with longer days of hospitalization (e.g., % difference: 273.1, 95% CI: 222.8, 331.3 for cases hospitalized over 28 days compared with cases hospitalized under 14 days). Higher costs were also found in severe cases (% difference: 344.8, 95% CI: 222.5, 513.6) than in mild cases, and in cases whose clinical conditions aggravated during hospitalization (% difference: 123.7, 95% CI: 92.6, 159.9) than cases without aggravation.

**Table 2:** Associated factors of the total hospitalization costs in COVID-19 cases.

**Characteristics**	**Median (IQR) (× $1000)**	**% Difference^a,b^ (95%CI)**
**Sex**		
Female	2.7 (3.3)	Reference
Male	3.2 (4.9)	29.7 (15.5, 45.6)
**Age (years)**		
0–19	1.7 (1.4)	Reference
20–29	2.1 (2.0)	44.0 (12.6, 84.1)
30–39	2.4 (2.3)	50.8 (20.3, 89.1)
40–49	2.6 (2.9)	82.0 (42.6, 132.4)
50–59	4.0 (4.6)	134.4 (85.6, 196.1)
60–69	6.3 (8.9)	280.6 (199.1, 384.3)
≥70	5.1 (9.7)	323.0 (202.9, 490.7)
Hospital level of admission		
Secondary hospital	1.8 (1.8)	Reference
Tertiary hospital	3.0 (4.1)	51.3 (22.8, 86.3)
Days of hospitalization		
0–14	1.5 (1.0)	Reference
15–21	2.6 (2.8)	62.5 (42.7, 85.1)
22–28	3.5 (4.3)	119.2 (88.6, 154.8)
>28	5.7 (8.7)	273.1 (222.8, 331.3)
Clinical severity at admission		
Mild	2.2 (1.8)	Reference
Moderate	2.8 (3.6)	8.8 (−11.0, 33.1)
Severe	15.1 (30.1)	344.8 (222.5, 513.6)
Clinical aggravation during hospitalization		
No	2.6 (2.7)	Reference
Yes	7.3 (10.0)	123.7 (92.6, 159.9)
Death during hospitalization		
No	2.8 (3.9)	Reference
Yes	162.9 (37.3)	1753.2 (697.4, 4207.2)
Clinical symptoms at admission		
No	2.1 (1.5)	Reference
Yes	3.2 (4.5)	47.7 (26.2, 72.9)
Fever		
No	2.3 (2.1)	Reference
Yes	3.3 (4.7)	42.1 (25.4, 61.1)
Cough		
No	2.7 (3.0)	Reference
Yes	3.1 (4.8)	14.6 (2.1, 28.7)
**Muscle pain**		
No	2.8 (3.7)	Reference
Yes	3.7 (5.6)	15.0 (−8.0, 43.8)
**Comorbidity**		
No	2.6 (2.9)	Reference
Yes	4.4 (6.3)	21.1 (4.4, 40.6)
Diabetes		
No	2.7 (3.7)	Reference
Yes	5.8 (11.2)	33.4 (2.3, 74.0)
Chronic kidney disease		
No	2.8 (3.8)	Reference
Yes	7.5 (10.5)	74.2 (14.2, 165.6)
Chronic lung disease		
No	2.8 (3.8)	Reference
Yes	4.2 (7.2)	11.2 (−15.9, 47.1)
Hypertension		
No	2.7 (3.4)	Reference
Yes	5.6 (9.2)	23.2 (0.7, 50.7)
Cardiovascular disease		
No	2.8 (3.6)	Reference
Yes	7.3 (9.9)	49.2 (7.3, 107.5)
Oxygen inhalation therapy		
No	3.6 (5.1)	Reference
Yes	2.2 (2.0)	41.1 (24.4, 60.0)
Oxyhydrogen atomizer therapy		
No	2.7 (3.6)	Reference
Yes	9.3 (6.1)	142.1 (72.7, 239.4)
Non-invasive ventilator therapy		
No	2.7 (3.1)	Reference
Yes	13.1 (13.6)	194.1 (137.8, 263.9)
Tracheal cannula therapy		
No	2.8 (3.6)	Reference
Yes	62.3 (109.9)	1142.3 (799.9, 1615.0)
Hormone usage		
No	2.7 (3.2)	Reference
Yes	13.8 (1.5)	298.6 (219.5, 397.5)

a *Adjusted for sex, age and hospital level of admission*.

b *The costs were calculated at the exchange rate between RMB and U.S dollars on August 1, 2020*.

**Table 3:** Associated factors of the total hospitalization costs in COVID-19 cases.

**Characteristics**	**Median (IQR) (× $1,000)**	**% Difference^a,d^ (95%CI)**
**ICU therapy^b^**		
No	2.7 (3.3)	Reference
Yes	19.0 (53.1)	353.0 (253.8, 479.9)
**Anti-infective drug usage**		
No	2.4 (2.2)	Reference
Yes	4.1 (6.4)	58.4 (40.8, 78.1)
**Antiviral drug usage**		
No	2.9 (4.2)	Reference
Yes	2.6 (1.4)	16.0 (−7.9, 46.0)
**ECMO therapy^c^**		
No	2.8 (3.7)	Reference
Yes	154.5 (88.3)	1524.8 (887.3, 2574.0)
**Angiotensin drugs usage**		
No	2.8 (3.7)	Reference
Yes	92.5 (137.6)	898.9 (562.8, 1405.5)
**Chloroquine phosphate usage**		
No	2.9 (4.0)	Reference
Yes	2.7 (0.6)	13.4 (−36.3, 102.1)

a *Adjusted for sex, age, and hospital level of admission*.

b *ICU, Intensive care unit*.

c *ECMO, Extracorporeal membrane oxygenation*.

d *The costs were calculated at the exchange rate between RMB and U.S dollars on August 1, 2020*.

Cases with symptoms at admission had 47.7% (95% CI: 26.2%, 72.9%) higher hospitalization costs than those without symptoms. In particular, cases with fever at admission had 42.1% (95% CI: 25.4%, 61.1%) higher hospitalization costs than those without fever. Compared with cases without comorbidities, cases with comorbidities had higher hospitalization costs (% difference: 21.1, 95% CI: 4.4, 40.6), particularly in cases with diabetes (% difference: 33.4, 95% CI: 2.3, 74.0), cardiovascular diseases (% difference: 49.2, 95% CI: 7.3, 107.5), and chronic kidney diseases (% difference: 74.2, 95% CI: 14.2, 165.5).

We also observed higher costs in cases who received oxygen inhalation therapy (% difference: 41.1, 95% CI: 24.4, 60.0), oxyhydrogen atomizer therapy (% difference: 142.1, 95% CI: 72.7, 239.4), non-invasive ventilator therapy (% difference: 194.1, 95% CI: 137.8, 263.9), tracheal cannula therapy (% difference: 1142.3, 95% CI: 799.9, 1615.0), extracorporeal membrane oxygenation (ECMO) therapy (% difference: 1524.8, 95% CI: 887.3, 2574.0), intensive care unit (ICU) therapy (% difference: 353.0, 95% CI: 253.8, 479.9), anti-infective drug (% difference: 58.4, 95% CI: 40.8, 78.1), angiotensin drugs (% difference: 898.9, 95% CI: 562.8, 1405.5), and hormone (% difference: 298.6, 95% CI: 219.5, 397.5) than cases who did not receive the corresponding therapy, respectively. Similar associated factors were found for drug usage costs, examination costs, and non-pharmacologic therapy costs (Supplementary Tables 2–4).

### The Associations of TCM With Drug Usage and Non-pharmacologic Therapy Costs

We observed significantly negative associations of TCM with non-pharmacologic therapy costs in the total cases (% difference: −47.4, 95% CI: −64.5 to −22.0), in moderate cases (% difference: −51.0, 95% CI: −69.1 to −22.3), and in cases without comorbidities (% difference: −49.1, 95% CI: −66.5 to −22.5). However, we did not find a significant association of TCM with drug usage costs (Table 4).

**Table 4:** Associations (% Difference, 95% CI) of drug usage and non-pharmacologic therapy costs with TCM therapy.

**Costs type**	**All cases^a^**	**Clinical severity at admission**		**Comorbidity^a^**		**Antiviral drugs usage^a^**	
		**Mild**	**Moderate**	**Yes**	**No**	**Yes**	**No**
**Drug usage costs**							
Non TCM therapy	Reference	Reference	Reference	Reference	Reference	Reference	Reference
TCM therapy	34.1 (−17.6, 118.4)	159.7 (−13.1, 675.6)	10.2 (−37.7, 95.1)	20.3 (−63.1, 292.2)	33.4 (−22.7, 130.1)	16.0 (−48.3, 160.3)	−8.1 (−89.4, 694.9)
**Non-pharmacologic therapy costs**							
Non TCM therapy	Reference	Reference	Reference	Reference	Reference	Reference	Reference
TCM therapy	−47.4 (−64.5 to −22.0)	−35.8 (−73.3 to 54.3)	−51.0 (−69.1 to −22.3)	−37.8 (−79.2, 86.1)	−49.1 (−66.5 to −22.5)	−43.5 (−77.2, 39.6)	−8.7 (−83.3, 400.6)

a *Adjusted for sex, age, hospital level of admission and clinical severity at admission*.

## Discussion

In this study, we investigated the total and components of hospitalization costs of COVID-19 cases in Guangdong province, China, and examined the associated factors of costs. We observed that the median of total hospitalization costs was $2,869.4, in which the median costs of drug usage, examination, and non-pharmacologic therapy were $631.3, $416.1, and $258.3, respectively. Factors that increase the hospitalization cost included male, older age, higher level of hospital, longer days of hospitalization, severe clinical conditions, clinical aggravation during hospitalization, clinical symptoms at admission, drug usage such as angiotensin drugs, and non-pharmacologic therapy ICU care, and ECMO therapy. Furthermore, the TCM therapy could reduce the non-pharmacologic costs. Our findings could help clinical doctors and health care managers understand the factors which influence the hospitalization costs, better allocate limited medical resources, and improve treatment strategies in early stage. In addition, we provided information for medical insurance department to determine reimbursement standards, make relevant policies, and optimize the utilization of medical sources.

The total hospitalization cost of COVID-19 cases in this study had a large variation, ranged from $0.4 million to $53.0. The case with the maximum cost of $0.4 million was a 74-year-old male clinical severe cases with hypertension, cardiovascular disease and chronic kidney disease, who was hospitalized for 37 days, and was treated with continuous renal replacement therapy (CRRT), ECMO, and ICU cares. By contrast, the case with the minimum cost of $53.1 was a 59-year-old male mild case without any comorbidity, who was discharged without any specific treatments. Although very few studies have reported that hospitalization costs of COVID-19 cases in China (4, 6), a national report showed that the mean hospitalization costs of COVID-19 cases were $3,084.6 (6), which is comparable to our findings ($2,869.4). This comparability may be partially explained by the similar distribution of clinical severity between COVID-19 cases in the present study and at nationwide. For example, the percentage of severe cases in this study was 17.0%, which was comparable to the national level (19.0% of severe rate) (16). These results further suggest the good representativeness of our study subjects.

We further found that the hospitalization costs of COVID-19 cases in this study were lower than in most countries globally (Supplementary Table 5). For example, the median of total cost was lower than that in the USA, India and Indonesia, and slightly higher than in Kenya (17–21). After categorizing COVID-19 cases by clinical severity, we found lower hospitalization costs in mild, moderate, and severe COVID-19 cases in this study than in South Korea and the USA, but higher than in Russia and in Kenya (21–24). The differences between China and other countries may be related to different level of development, policy, drug, and non-pharmacologic therapy costs, and therapeutic regimes. Since the very early stage of COVID-19 epidemic, inspection teams consisting of academicians and experts were organized to regularly inspect designated hospitals and discuss and evaluate the treatment plans for COVID-19 cases. The diagnosis and treatment protocols of COVID-19 cases were constantly improved, which has substantially declined the hospitalization costs of COVID-19 cases in the late period of epidemic (Figure 2), and set an example in effective treatment of COVID-19 cases. In contrast, higher hospitalization costs were found before February 14, 2020 (22 out of 796 cases had more than $30,000 hospitalization costs, and the highest costs was more than $430.0 thousand during this period). The probable causes might be related to the poorer understanding on SARS-CoV-2 virus and having no specific clinical therapies in the early period of epidemic of the COVID-19. Some cases might be delayed care during this period, which would lead to the aggravation of the illness, and substantially increase the hospitalization costs because those severe cases are more likely to use expensive therapy such as ECMO.

As we expected, significantly higher hospitalization costs were observed in severe cases than in mild or moderate cases, and in cases with clinical aggregation during hospitalization. Previous studies have reported that severe and critically severe cases had higher risks of clinical aggregation, multiple organ failure, and fatality (25, 26), which was associated with higher medical costs. To maximally improve the cure rate of COVID-19 cases, all severe cases in China were treated following the principle: on the basis of symptomatic treatment, complications should be proactively prevented, comorbidities should be treated, secondary infections also be prevented, and organ function support should be provided timely (13). Based on the principle, severe cases were more frequently treated with high-flow nasal-catheter oxygenation, non-invasive mechanical ventilation, ICU care, ECMO, and CRRT, which hence increase their hospitalization costs. In this study, we also found higher costs in cases treated with ECMO, tracheal cannula, non-invasive ventilator, oxyhydrogen atomizer, and oxygen inhalation therapy.

We also found higher hospitalization costs in cases with clinical symptoms particularly for fever at admission than those without symptoms. Cases with symptoms usually need more support therapy, antiviral and antibiotic therapy during their hospitalization (Supplementary Table 6), which can create additional costs. Both previous studies and the present study have found that clinical symptoms such as fever were common in admitted COVID-19 cases (27–29). Thus, the symptoms at admission, especially fever, can be used as one of the important predictors of hospitalization costs because of the high proportion in clinical features.

Many studies reported that the comorbidities such as hypertension, diabetes, cardiovascular disease, and respiratory diseases could greatly affect the prognosis and mortality of the COVID-19 (25, 30–32). For example, a meta-analysis including 13 studies found that cases with hypertension had 1.72 times higher critical/mortal risk than those without hypertension (31). Therefore, it is reasonable to hypothesize that cases with comorbidities may have higher hospitalization costs than those without comorbidities. As expected, our findings found higher costs in cases with comorbidities at admission. These cases had higher proportions of severe conditions, and clinical aggravation during hospitalization, resulting in the increase of their hospitalization costs (Supplementary Table 7). This finding also indicates that comorbidities could also be used as predictors of hospitalization costs for COVID-19 cases.

TCM has a long history and played an indispensable role in the prevention and treatment of several epidemic diseases in China, such as severe acute respiratory syndrome (SARS) (33). TCM scheme was included in the guideline on diagnosis and treatment of COVID-19 cases as a major feature in China (13). It was widely used in patients with mild symptoms, and was also used in combination with western medicines in patients with severe symptoms. It was reported that more than 90% of COVID-19 cases both in Hubei and the rest of China were treated with TCM (34). In this study, we observed significantly negative associations of TCM usage with non-pharmacologic therapy costs in the total cases, moderate cases, and in cases without comorbidities. Previous studies showed that TCM strategies showed apparent advantages in improving symptoms, promoting virus clearance, increasing cure rate, shortening hospitalization, and reducing patient progression from mild to severe (9, 35, 36). These findings suggest the potential of TCM in saving hospitalization costs in COVID-19 cases, although dedicated and more rigorous studies are desirable before wide application of TCM for treatment of COVID-19 cases to other countries.

In addition, we found higher hospitalization costs in male cases than in female cases, which may be related to the sex differences in genes and health status. Studies have reported that male COVID-19 cases had higher prevalence of comorbidities (31), and hence had greater risk of severer clinical condition and mortality than female COVID-19 cases (25, 31, 32, 37, 38). Moreover, X chromosome and sex hormones in females play an important role in innate and adaptive immunity, which could protect them from clinical aggregation (39). The higher cost in older cases was related to their higher risk of COVID-19 infection, morbidity, aggregation, and death (25, 31, 40, 41). Older cases often have comorbidities like hypertension, diabetes and cardiovascular disease, and as the body's immunity declines with age, they are more likely to develop critical illness or even die, which also increase their hospitalization costs.

### Strengths and Limitations

This study has several strengths. First, this study provides the total and components of hospitalization costs of COVID-19 cases in China. This result could provide clear information for systematically evaluating the direct economic burden of COVID-19 in China and worldwide. Second, we investigated associated factors of hospitalization costs including demographic characteristics, clinical severity, clinical symptoms, comorbidities, and specific clinical therapies, which could help clinical doctors to make treatment strategies, and medical insurance department to determine reimbursement standards. Third, we estimated the associations between TCM and hospitalization costs, which provides more evidence for the potential of TCM in saving costs for COVID-19 cases.

Several limitations also should be noted. First, we only obtained the information of COVID-19 cases in Guangdong Province, China, and the 876 cases' dataset was relatively small, which may limit the generalization of our findings. However, the median of total hospitalization cost in this study was comparable to the national level, which may indicate the good representativeness of our findings. Second, the costs information was from the designed hospital where the cases stayed at the time of recruitment, and costs incurred in other hospitals due to hospital transfer and other reasons were not included in this study, which may lead potential information bias. Third, hospitalization costs of COVID-19 cases in other countries were mainly obtained from news or report. The information was not peer reviewed, and may provide biased information.

## Conclusions

We observed a comparable hospitalization costs of COVID-19 cases in Guangdong with the national level. Factors leading to higher hospitalization costs included sex, older age, clinical severity and aggravation, clinical symptoms and comorbidities at admission, drug usage, and some non-pharmacologic therapies. In addition, TCM therapy may reduce non-pharmacologic therapy costs in mild and moderate cases. Our findings have both clinical and public health implications for containing the spread of COVID-19.

## Data Availability Statement

The original contributions presented in the study are included in the article/Supplementary Material, further inquiries can be directed to the corresponding authors.

## Ethics Statement

This study was approved by the Ethics Committee of Guangdong Provincial Center for Disease Control and Prevention (No. W96-027E-2020004). The data analysis was carried out at a population level after data aggregation. We didn't contact any individual subjects.

## Author Contributions

TL and MF designed the study, collected and standardized the data, and coordinated the work. MD and ZY performed the statistical analysis and drafted the manuscript and interpreted the results. TL provided substantial scientific insight into the interpretation of the results and drafting of the manuscript. YC, JS, WM, SC XS, JX, GH, JH, JW, GC, HZ, LY, JL, HX, XL, DC, and RW provided the data, and contributed to the interpretation of the results and the preparation of the submitted version of the manuscript. All authors contributed to the article and approved the submitted version.

## Conflict of Interest

The authors declare that the research was conducted in the absence of any commercial or financial relationships that could be construed as a potential conflict of interest.
