# Supplementary material for: Hospitalization Costs of COVID-19 Cases and Their Associated Factors in Guangdong, China: A Cross-Sectional Study
Source: Front Med (Lausanne). 2021 Jun 11;8:655231. doi: 10.3389/fmed.2021.655231 (PMC8226137; doi:10.3389/fmed.2021.655231)
Supplement: Supplementary file 1 [file Table_1.DOCX]

**Supplementary materials**

**Hospitalization costs of COVID-19 cases and their associated factors in Guangdong, China: A cross-sectional study**

Appendix

[Table S1. Detailed information of hospitalization costs 1](#_Toc61187398)

[Table S2. Associated factors of the drug usage costs in COVID-19 cases 2](#_Toc61187399)

[Table S3. Associated factors of the examination costs in COVID-19 cases 4](#_Toc61187400)

[Table S3. Associated factors of the examination costs in COVID-19 cases (*continued*) 5](#_Toc61187401)

[Table S4. Associated factors of the nonpharmacologic therapy costs in COVID-19 cases 6](#_Toc61187402)

[Table S4. Associated factors of the nonpharmacologic therapy costs in COVID-19 cases (*continued*) 7](#_Toc61187403)

[Table S5. Comparison of hospitalization costs of COVID-19 cases between China and other countries 8](#_Toc61187404)

[Table S6. Differences in drug usage and nonpharmacologic therapies between COVID-19 cases with and without symptoms at admission 10](#_Toc61187405)

[Table S7. Differences in clinical severity at admission and disease progression between COVID-19 cases with and without comorbidity at admission 11](#_Toc61187406)

Table S1. Detailed information of hospitalization costs

|  | Definitions |
| --- | --- |
| Drug usage costs | The costs of all drugs used during hospitalization, including western medicines and Traditional Chinese Medicines. |
| Examination costs | Diagnosis related examination costs, including ultrasound, radiation imaging and clinical test, *etc*. |
| Nonpharmacologic therapy costs | The costs of relevant operations manipulated by health care workers, including nursing, oxygen inhalation, injection, dressing change, stomach tube, catheterization, surgical operation, ventilator, ECMO ^a^ and CRRT, *etc.* |
| Other costs | Other costs including the costs of general diagnosing, supervision, first aid, physical examination, bed and consultation, *etc*. |
| Total costs | All costs during the hospitalization of a COVID-19 case, including drug usage costs, examination costs, nonpharmacologic therapy costs, and other costs. |

a ECMO: Extracorporeal membrane oxygenation; CRRT: Continuous renal replacement therapy

Table S2. Associated factors of the drug usage costs in COVID-19 cases

|  | Median (IQR)  (× $1000) | % Difference ^a, c^  (95%CI) |  | Median (IQR)  (× $1000) | % Difference ^a, c^  (95%CI) |
| --- | --- | --- | --- | --- | --- |
| Sex |  |  | Muscle pain |  |  |
| Female | 0.6 (1.2) | Reference | No | 0.6 (2.3) | Reference |
| Male | 0.6 (2.7) | 41.2 (13.2, 76.1) | Yes | 1.5 (4.0) | 36.6 (-9.6, 106.2) |
| Age (years) |  |  | Comorbidity |  |  |
| 0-19 | 0.2 (0.4) | Reference | No | 0.5 (1.7) | Reference |
| 20-29 | 0.4 (0.8) | 127.8 (42.5, 264.1) | Yes | 1.4 (4.3) | 38.4 (4.4, 83.3) |
| 30-39 | 0.4 (1.1) | 151.0 (62.4, 287.9) | Diabetes |  |  |
| 40-49 | 0.6 (2.2) | 257.3 (123.7, 470.5) | No | 0.6 (2.3) | Reference |
| 50-59 | 1.4 (3.4) | 494.7 (279.7, 831.6) | Yes | 3.2 (6.5) | 103.8 (23.0, 237.8) |
| 60-69 | 2.8 (5.4) | 1006.2 (598.6, 1651.7) | Chronic kidney disease |  |  |
| $\geq$70 | 2.3 (5.5) | 1096.7 (523.5, 2197.0) | No | 0.6 (2.5) | Reference |
| Hospital level of admission |  |  | Yes | 1.9 (5.1) | 61.2 (-29.4, 268) |
| Secondary hospital | 0.6 (1.2) | Reference | Chronic lung disease |  |  |
| Tertiary hospital | 0.6 (2.7) | -0.2 (-32.0, 46.6) | No | 0.6 (2.5) | Reference |
| Days of hospitalization |  |  | Yes | 1.2 (4.3) | 24.3 (-26.2, 109.5) |
| 0-14 | 0.3 (0.4) | Reference | Hypertension |  |  |
| 15-21 | 0.5 (2.0) | 122.8 (68.9, 193.8) | No | 0.6 (2.0) | Reference |
| 22-28 | 1.0 (2.9) | 239.1 (147.6, 364.6) | Yes | 1.9 (5.2) | 16.2 (-20.7, 70.3) |
| >28 | 1.9 (5.0) | 496.7 (340.3, 708.6) | Cardiovascular disease |  |  |
| Clinical severity at admission |  |  | No | 0.6 (2.4) | Reference |
| Mild | 0.3 (0.7) | Reference | Yes | 3.0 (5.8) | 24.2 (-34.4, 134.9) |
| Moderate | 0.6 (2.2) | 81.2 (23.1, 166.7) | Oxygen inhalation therapy |  |  |
| Severe | 5.9 (10.0) | 958.8 (470.8, 1863.9) | No | 0.4 (0.8) | Reference |
| Clinical aggravation during hospitalization |  |  | Yes | 1.2 (3.6) | 73.2 (36.1, 120.5) |
| No | 0.5 (1.4) | Reference | Oxyhydrogen atomizer therapy |  |  |
| Yes | 3.6 (6.6) | 229.4 (147.5, 338.3) | No | 0.6 (2.4) | Reference |
| Death during hospitalization |  |  | Yes | 3.7 (3.6) | 217.0 (67.4, 500.0) |
| No | 0.6 (2.5) | Reference | Noninvasive ventilator therapy |  |  |
| Yes | 61.9 (16.6) | 2222.3 (380.2, 11130.1) | No | 0.5 (1.6) | Reference |
| Clinical symptoms at admission |  |  | Yes | 5.9 (6.3) | 438.8 (261.8, 702.3) |
| No | 0.3 (0.6) | Reference | Tracheal cannula therapy |  |  |
| Yes | 0.8 (2.9) | 142.3 (78.2, 229.4) | No | 0.6 (2.2) | Reference |
| Fever |  |  | Yes | 28.8 (41.6) | 1684.8 (813.7, 3386.4) |
| No | 0.3 (0.9) | Reference | ECMO therapy ^b^ |  |  |
| Yes | 0.9 (3.2) | 119.6 (72.8, 179.2) | No | 0.6 (2.4) | Reference |
| Cough |  |  | Yes | 60.5 (37.8) | 1509.4 (455.2, 4565.2) |
| No | 0.5 (1.7) | Reference |  |  |  |
| Yes | 0.8 (3.2) | 34.2 (7.6, 67.4) |  |  |  |

a Adjusted for sex, age and hospital level of admission

b ECMO: Extracorporeal membrane oxygenation

c The costs were calculated at the exchange rate between RMB and US dollars on August 1, 2020.

Table S2. Associated factors of the drug usage costs in COVID-19 cases (*continued*)

|  | Median (IQR)  (× $1000) | % Difference ^a, c^  (95%CI) |  | Median (IQR)  (× $1000) | % Difference ^a, c^  (95%CI) |
| --- | --- | --- | --- | --- | --- |
| ICU therapy ^b^ |  |  | Angiotensin drugs usage |  |  |
| No | 0.5 (1.9) | Reference | No | 0.6 (2.3) | Reference |
| Yes | 8.5 (21.6) | 566.9 (313.1, 976.5) | Yes | 40.6 (53.2) | 1440.5 (568.7, 3448.7) |
| Anti-infective drug usage |  |  | Hormone usage |  |  |
| No | 0.4 (1.0) | Reference | No | 0.5 (1.7) | Reference |
| Yes | 1.4 (4.0) | 121.0 (76.5, 176.7) | Yes | 6.8 (6.9) | 593.6 (354.9, 957.6) |
| Antiviral drug usage |  |  | Chloroquine phosphate usage |  |  |
| No | 0.6 (2.7) | Reference | No | 0.6 (2.6) | Reference |
| Yes | 0.5 (0.8) | 3.1 (-32.8, 58.1) | Yes | 0.8 (0.9) | -19.1 (-72.0, 134.0) |

a Adjusted for sex, age and hospital level of admission.

b ICU: Intensive care unit.

c The costs were calculated at the exchange rate between RMB and US dollars on August 1, 2020.

Table S3. Associated factors of the examination costs in COVID-19 cases

|  | Median (IQR)  (× $1000) | % Difference ^a, c^  (95%CI) |  | Median (IQR)  (× $1000) | % Difference ^a, c^  (95%CI) |
| --- | --- | --- | --- | --- | --- |
| Sex |  |  | Muscle pain |  |  |
| Female | 0.4 (0.7) | Reference | No | 0.4 (0.8) | Reference |
| Male | 0.5 (0.9) | 41.2 (13.2, 76.1) | Yes | 0.6 (0.9) | 18.4 (-8.3, 52.8) |
| Age (years) |  |  | Comorbidity |  |  |
| 0-19 | 0.3 (0.4) | Reference | No | 0.4 (0.7) | Reference |
| 20-29 | 0.3 (0.7) | 127.8 (42.5, 264.1) | Yes | 0.5 (1.3) | 12.7 (-5.4, 34.2) |
| 30-39 | 0.3 (0.5) | 151 (62.4, 287.9) | Diabetes |  |  |
| 40-49 | 0.3 (0.6) | 257.3 (123.7, 470.5) | No | 0.4 (0.7) | Reference |
| 50-59 | 0.5 (1.0) | 494.7 (279.7, 831.6) | Yes | 0.9 (1.9) | 44.2 (4.7, 98.7) |
| 60-69 | 0.8 (1.7) | 1006.2 (598.6, 1651.7) | Chronic kidney disease |  |  |
| $\geq$70 | 0.8 (1.9) | 1096.7 (523.5, 2197.0) | No | 0.4 (0.8) | Reference |
| Hospital level of admission |  |  | Yes | 1.0 (2.2) | 60.4 (-3.8, 167.5) |
| Secondary hospital | 0.7 (0.3) | Reference | Chronic lung disease |  |  |
| Tertiary hospital | 0.4 (0.9) | -0.2 (-32, 46.6) | No | 0.4 (0.8) | Reference |
| Days of hospitalization |  |  | Yes | 0.5 (2.0) | 14.5 (-17.2, 58.4) |
| 0-14 | 0.3 (0.5) | Reference | Hypertension |  |  |
| 15-21 | 0.3 (0.7) | 27.8 (7.1, 52.6) | No | 0.4 (0.7) | Reference |
| 22-28 | 0.4 (0.6) | 50.1 (22.8, 83.6) | Yes | 0.8 (1.8) | 25.1 (-1.3, 58.7) |
| >28 | 0.9 (1.7) | 149.4 (105.4, 202.9) | Cardiovascular disease |  |  |
| Clinical severity at admission |  |  | No | 0.4 (0.7) | Reference |
| Mild | 0.4 (0.7) | Reference | Yes | 1.2 (1.2) | 28.2 (-14.2, 91.7) |
| Moderate | 0.4 (0.7) | 4.1 (-18.3, 32.7) | Oxygen inhalation therapy |  |  |
| Severe | 1.7 (2.0) | 185.2 (93.6, 320.2) | No | 0.3 (0.8) | Reference |
| Clinical aggravation during hospitalization |  |  | Yes | 0.5 (0.8) | 16.1 (-0.2, 35.1) |
| No | 0.3 (0.6) | Reference | Oxyhydrogen atomizer therapy |  |  |
| Yes | 0.9 (1.8) | 87.7 (56.8, 124.6) | No | 0.4 (0.8) | Reference |
| Death during hospitalization |  |  | Yes | 0.6 (0.5) | 10.4 (-25.9, 64.5) |
| No | 0.4 (0.8) | Reference | Noninvasive ventilator therapy |  |  |
| Yes | 5.1 (6.7) | 659.7 (186.0, 1917.9) | No | 0.4 (0.7) | Reference |
| Clinical symptoms at admission |  |  | Yes | 1.2 (1.4) | 102.2 (56.8, 160.7) |
| No | 0.3 (0.5) | Reference | Tracheal cannula therapy |  |  |
| Yes | 0.5 (0.8) | 29.8 (6.9, 57.5) | No | 0.4 (0.7) | Reference |
| Fever |  |  | Yes | 3.1 (3.3) | 376.5 (213.2, 625.0) |
| No | 0.3 (0.7) | Reference | ECMO therapy ^b^ |  |  |
| Yes | 0.5 (0.8) | 11.4 (-4.4, 29.8) | No | 0.4 (0.8) | Reference |
| Cough |  |  | Yes | 3.7 (4.0) | 360.7 (137.5, 793.7) |
| No | 0.4 (0.7) | Reference |  |  |  |
| Yes | 0.5 (1.0) | 16.5 (1.6, 33.7) |  |  |  |

a Adjusted for sex, age and hospital level of admission

b ECMO: Extracorporeal membrane oxygenation

c The costs were calculated at the exchange rate between RMB and US dollars on August 1, 2020.

Table S3. Associated factors of the examination costs in COVID-19 cases (*continued*)

|  | Median (IQR)  (× $1000) | % Difference ^a, c^  (95%CI) |  | Median (IQR)  (× $1000) | % Difference ^a, c^  (95%CI) |
| --- | --- | --- | --- | --- | --- |
| ICU therapy ^b^ |  |  | Angiotensin drugs usage |  |  |
| No | 0.4 (0.7) | Reference | No | 0.4 (0.7) | Reference |
| Yes | 2.0 (2.6) | 199.8 (122.4, 304.2) | Yes | 4.1 (4.3) | 395.8 (195.0, 733.5) |
| Anti-infective drug usage |  |  | Hormone usage |  |  |
| No | 0.3 (0.3) | Reference | No | 0.4 (0.7) | Reference |
| Yes | 0.8 (1.6) | 127.6 (99.5, 159.8) | Yes | 1.3 (1.7) | 121.6 (69.3, 190.0) |
| Antiviral drug usage |  |  | Chloroquine phosphate usage |  |  |
| No | 0.4 (0.8) | Reference | No | 0.4 (0.8) | Reference |
| Yes | 0.4 (0.6) | -2.5 (-25.2, 27.2) | Yes | 0.9 (0.9) | 118.1 (13.1, 320.5) |

a Adjusted for sex, age and hospital level of admission.

b ICU: Intensive care unit.

c The costs were calculated at the exchange rate between RMB and US dollars on August 1, 2020.

Table S4. Associated factors of the nonpharmacologic therapy costs in COVID-19 cases

|  | Median (IQR)  (× $1000) | % Difference ^a, c^  (95%CI) |  | Median (IQR)  (× $1000) | % Difference ^a, c^  (95%CI) |
| --- | --- | --- | --- | --- | --- |
| Sex |  |  | Muscle pain |  |  |
| Female | 0.2 (0.3) | Reference | No | 0.2 (0.4) | Reference |
| Male | 0.3 (0.5) | 29.2 (7.4, 55.3) | Yes | 0.3 (0.5) | 36.5 (-3.1, 92.4) |
| Age (years) |  |  | Comorbidity |  |  |
| 0-19 | 0.1 (0.1) | Reference | No | 0.2 (0.3) | Reference |
| 20-29 | 0.2 (0.2) | 108.9 (41.3, 208.8) | Yes | 0.3 (0.8) | 15.6 (-8.6, 46.2) |
| 30-39 | 0.2 (0.3) | 92.9 (34.2, 177.3) | Diabetes |  |  |
| 40-49 | 0.3 (0.3) | 204.6 (106.2, 349.9) | No | 0.2 (0.4) | Reference |
| 50-59 | 0.3 (0.4) | 321.8 (190.2, 513.1) | Yes | 0.5 (1.4) | 57.2 (3.1, 139.7) |
| 60-69 | 0.5 (1.1) | 559.9 (349.9, 867.9) | Chronic kidney disease |  |  |
| $\geq$70 | 0.5 (1.0) | 772.1 (406.4, 1401.7) | No | 0.2 (0.4) | Reference |
| Hospital level of admission |  |  | Yes | 0.8 (1.1) | 91.5 (-3.6, 280.7) |
| Secondary hospital | 0.3 (0.4) | Reference | Chronic lung disease |  |  |
| Tertiary hospital | 0.2 (0.4) | -17.2 (-39.9, 14.1) | No | 0.2 (0.4) | Reference |
| Days of hospitalization |  |  | Yes | 0.4 (1.0) | 26.5 (-18.1, 95.3) |
| 0-14 | 0.1 (0.2) | Reference | Hypertension |  |  |
| 15-21 | 0.3 (0.3) | 46.4 (15.9, 84.8) | No | 0.2 (0.4) | Reference |
| 22-28 | 0.4 (0.4) | 85.2 (42.0, 141.5) | Yes | 0.4 (1.1) | 21.3 (-11.8, 66.7) |
| >28 | 0.5 (1.1) | 253 (173.2, 356.1) | Cardiovascular disease |  |  |
| Clinical severity at admission |  |  | No | 0.2 (0.4) | Reference |
| Mild | 0.1 (0.2) | Reference | Yes | 0.9 (1.4) | 48.4 (-12.7, 152.3) |
| Moderate | 0.2 (0.4) | 93.8 (41.5, 165.4) | Oxygen inhalation therapy |  |  |
| Severe | 1.6 (4.1) | 1166.6 (666.5, 1993.0) | No | 0.1 (0.1) | Reference |
| Clinical aggravation during hospitalization |  |  | Yes | 0.4 (0.5) | 256.6 (196.8, 328.5) |
| No | 0.2 (0.3) | Reference | Oxyhydrogen atomizer therapy |  |  |
| Yes | 0.6 (1.2) | 144.8 (92.6, 211.2) | No | 0.2 (0.4) | Reference |
| Death during hospitalization |  |  | Yes | 0.7 (0.8) | 135.0 (38.0, 300.4) |
| No | 0.2 (0.4) | Reference | Noninvasive ventilator therapy |  |  |
| Yes | 24.6 (4.8) | 3691.2 (931.2, 13838.5)) | No | 0.2 (0.3) | Reference |
| Clinical symptoms at admission |  |  | Yes | 1.3 (2.6) | 359.2 (230.3, 538.2) |
| No | 0.1 (0.2) | Reference | Tracheal cannula therapy |  |  |
| Yes | 0.2 (0.4) | 184.1 (121.1, 265.2) | No | 0.2 (0.4) | Reference |
| Fever |  |  | Yes | 7.6 (12.1) | 1837.1 (1023.2, 3240.5) |
| No | 0.1 (0.3) | Reference | ECMO therapy ^b^ |  |  |
| Yes | 0.3 (0.5) | 97.1 (61.4, 140.6) | No | 0.2 (0.4) | Reference |
| Cough |  |  | Yes | 15.7 (12.2) | 1888.3 (726.9, 4680.9) |
| No | 0.2 (0.3) | Reference |  |  |  |
| Yes | 0.3 (0.5) | 46.2 (21.8, 75.6) |  |  |  |

a Adjusted for sex, age and hospital level of admission

b ECMO: Extracorporeal membrane oxygenation

c The costs were calculated at the exchange rate between RMB and US dollars on August 1

Table S4. Associated factors of the nonpharmacologic therapy costs in COVID-19 cases (*continued*)

|  | Median (IQR)  (× $1000) | % Difference ^a, c^  (95%CI) |  | Median (IQR)  (× $1000) | % Difference ^a, c^  (95%CI) |
| --- | --- | --- | --- | --- | --- |
| ICU therapy ^b^ |  |  | Angiotensin drugs usage |  |  |
| No | 0.2 (0.3) | Reference | No | 0.2 (0.4) | Reference |
| Yes | 2.4 (5.8) | 586.5 (364, 915.7) | Yes | 15.2 (17.3) | 1675.3 (795.1, 3421.2) |
| Anti-infective drug usage |  |  | Hormone usage |  |  |
| No | 0.2 (0.3) | Reference | No | 0.2 (0.3) | Reference |
| Yes | 0.4 (0.7) | 100.4 (66.3, 141.6) | Yes | 1.7 (3.1) | 501.7 (325.1, 751.6) |
| Antiviral drug usage |  |  | Chloroquine phosphate usage |  |  |
| No | 0.3 (0.4) | Reference | No | 0.3 (0.4) | Reference |
| Yes | 0.1 (0.2) | -49.2 (-64.3 to -27.7) | Yes | 0.2 (0.2) | 25.9 (-48.0, 205) |

a Adjusted for sex, age and hospital level of admission

b ICU: Intensive care unit

c The costs were calculated at the exchange rate between RMB and US dollars on August 1, 2020.

Table S5. Comparison of hospitalization costs of COVID-19 cases between China and other countries

|  | Hospitalization costs ^a^ | Reference | Note |
| --- | --- | --- | --- |
| Hospitalization costs in overall cases |  |  |  |
| Guangdong, China | 19,822 yuan ($2,843) | This article | Median costs (mean hospital stay was 22 days) |
| Shenzhen, China | 12,289 yuan ($1,762) | (1) | Mean hospital stay was 11 days, 105 cases |
| China | 21,500 yuan ($3,083) | (2) |  |
| India | RS280,000- 350,000 ($3,640- 4,550) | (3) | 14-day treatment |
| USA | About $30,000 | (4) |  |
| USA | $9,763 | (5) | without complications |
|  | $20,292 | (5) | With complications |
| Indonesia | IDR 105,000,000- 23,100,000 ($7,226-15,898) | (6) | Hospitalization for 14 days treatment |
| Kenya | Sh300,000 ($2,787) | (7) | Hospitalization for 14 days treatment |
| Johannesburg, South Africa | ZAF16,000 ($941) per day | (8) |  |
| Hospitalization costs in mild cases |  |  |  |
| Guangdong, China | 15,131 yuan ($2,170) | This article | Median costs (mean hospital stay was 23 days) |
| South Korea | KRW 3,310,000-4,780,000 ($2,775-4,007) | (9) |  |
| Russia | About RUB 70,000 ($943) | (10) |  |
| Japan | JPY 500,000 ($4,722) | (11) | Median costs |
| Kenya | Sh21,400 ($199) per day | (7) |  |
| Hospitalization costs in moderate cases |  |  |  |
| Guangdong, China | 19,310 yuan ($2,769) | This article | Median costs (mean hospital stay was 21 days) |
| USA | About $15,956 | (12) |  |
| Russia | About RUB 100,000 ($1,347) | (10) |  |
| Japan | JPY 500,000 ($4,722) | (11) | Median costs |
| South Korea | KRW 12,000,000 ($10,059) | (9) | Moderate and sever cases |
| Hospitalization costs in severe cases |  |  |  |
| Guangdong, China | 105,360 yuan ($15,109) | This article | Median costs (mean hospital stay was 31 days) |
| China | More than 150,000 yuan ($21,500) | (2) |  |
| USA | About $48,262 | (12) | Require more than 96 hours of ventilator support |
| Russia | More than RUB 200,000 ($2,695) | (10) | Without ECMO treatment |
| Germany | About EUR 100,000 ($117,740) | (13) | From France, Italy, Netherlands and other EU countries |
| Japan | JPY 600,000 ($5,667) | (11) | Median costs |
| Kenya | Sh 51,000 – 71,000 ($474 - $660) per day | (7) | Require supplemental oxygen fork |
| South Korea | KRW 12,000,000 - 70,000,000 ($10,059 - $58,676) | (9) | Moderate and sever cases |
| Drug usage costs in overall cases |  |  |  |
| Guangdong, China | 4,401 yuan ($631) | This article |  |
| India | RS 7,000 -14,000 ($112-182) | (3) | Antibiotics, anti-vitriol and other medicines for 14 days treatment |
| USA | $5,460 | (14) | 5 days Remdesivir treatment (without insurance) |
|  | $10,010 | (14) | 10 days Remdesivir treatment (without insurance) |

a The costs were calculated at the exchange rate between RMB and US dollars on August 1, 2020.

Table S5. Comparison of hospitalization costs of COVID-19 cases between China and other countries (*continued*)

|  | Hospitalization costs ^a^ | Reference | Note |
| --- | --- | --- | --- |
| Examination costs in overall cases |  |  |  |
| Guangdong, China | 2,900 yuan ($416) | This article |  |
| India | RS3,000 -4,500 ($39-59) | (3) | test kit alone / swab test |
| California, USA | $1,331 | (15) | Thoroughly COVID-19 test |
| Miami, USA | $3,270 | (16) | Emergency examination costs |

a The costs were calculated at the exchange rate between RMB and US dollars on August 1, 2020.

Table S6. Differences in drug usage and nonpharmacologic therapies between COVID-19 cases with and without symptoms at admission

|  | Without symptoms  N (%) | With symptoms  N (%) | $\chi$*^2^* | *P* |  | Without fever  N (%) | With fever  N (%) | $\chi$*^2^* | *P* |
| --- | --- | --- | --- | --- | --- | --- | --- | --- | --- |
| Oxygen inhalation therapy |  |  |  |  |  |  |  |  |  |
| Yes | 51 (35.7) | 482 (65.8) | 44.23 | <0.001 |  | 125 (45.1) | 408 (68.1) | 41.05 | <0.001 |
| No | 92 (64.3) | 251 (34.2) |  |  |  | 152 (54.9) | 191 (31.9) |  |  |
| Oxyhydrogen atomizer therapy |  |  |  |  |  |  |  |  |  |
| Yes | 0 (0) | 26 (3.5) | a | 0.014 |  | 3 (1.1) | 23 (3.8) | a | 0.043 |
| No | 143 (100) | 707 (96.5) |  |  |  | 274 (98.9) | 576 (96.2) |  |  |
| Noninvasive ventilator therapy |  |  |  |  |  |  |  |  |  |
| Yes | 0 (0) | 69 (9.4) | 13.34 | <0.001 |  | 6 (2.2) | 63 (10.5) | 17.07 | <0.001 |
| No | 143 (100) | 664 (90.6) |  |  |  | 271 (97.8) | 536 (89.5) |  |  |
| ICU therapy |  |  |  |  |  |  |  |  |  |
| Yes | 0 (0) | 49 (6.7) | 8.90 | 0.003 |  | 6 (2.2) | 43 (7.2) | 8.09 | 0.004 |
| No | 143 (100) | 684 (93.3) |  |  |  | 271 (97.8) | 556 (92.8) |  |  |
| TCM therapy |  |  |  |  |  |  |  |  |  |
| Yes | 16 (11.2) | 29 (4.0) | 11.40 | 0.001 |  | 23 (8.3) | 22 (3.7) | 7.41 | 0.004 |
| No | 127 (88.8) | 704 (96.0) |  |  |  | 254 (91.7) | 577 (96.3) |  |  |
| Antiviral drug usage |  |  |  |  |  |  |  |  |  |
| Yes | 22 (15.4) | 42 (5.7) | 15.08 | <0.001 |  | 34 (12.3) | 30 (5.0) | 13.71 | <0.001 |
| No | 121 (84.6) | 691 (94.3) |  |  |  | 243 (87.7) | 569 (95.0) |  |  |
| Anti-infective drug usage |  |  |  |  |  |  |  |  |  |
| Yes | 39 (27.3) | 349 (47.6) | 19.25 | <0.001 |  | 91 (32.9) | 297 (49.6) | 20.82 | <0.001 |
| No | 104 (72.7) | 384 (52.4) |  |  |  | 186 (67.1) | 302 (50.4) |  |  |

a Fisher's exact test.

Table S7. Differences in clinical severity at admission and disease progression between COVID-19 cases with and without comorbidity at admission

|  | Clinical severity at admission | | | |  | Death during hospitalization | | | | | Aggravation during hospitalization | | | |
| --- | --- | --- | --- | --- | --- | --- | --- | --- | --- | --- | --- | --- | --- | --- |
|  | Non-severe ^b^  N (%) | Severe  N (%) | $\chi$*^2^* | *P* |  | No  N (%) | Yes  N (%) | $\chi$*^2^* | *P* |  | No  N (%) | Yes  N (%) | $\chi$*^2^* | *P* |
| Comorbidity |  |  |  |  |  |  |  |  |  |  |  |  |  |  |
| Yes | 195 (87.8) | 27 (12.2) | 31.47 | <0.001 |  | 220 (99.1) | 2 (0.1) | a | 0.27 |  | 154 (69.4) | 82 (30.6) | 36.97 | <0.001 |
| No | 638 (97.6) | 16 (2.4) |  |  |  | 652 (99.7) | 2 (0.3) |  |  |  | 572 (87.5) | 68 (12.5) |  |  |
| Diabetes |  |  |  |  |  |  |  |  |  |  |  |  |  |  |
| Yes | 38 (82.6) | 8 (17.4) | a | 0.001 |  | 46 (100) | 0 (0) | a | 1.00 |  | 26 (56.5) | 20 (43.5) | 21.84 | <0.001 |
| No | 795 (95.8) | 35 (4.2) |  |  |  | 826 (99.5) | 4 (0.5) |  |  |  | 700 (84.3) | 130 (15.7) |  |  |
| Chronic kidney disease |  |  |  |  |  |  |  |  |  |  |  |  |  |  |
| Yes | 13 (76.5) | 4 (23.5) | a | 0.008 |  | 17 (100) | 0 (0) | a | 1.00 |  | 11 (64.7) | 6 (35.3) | a | 0.055 |
| No | 820 (95.5) | 39 (4.5) |  |  |  | 855 (99.5) | 4 (0.5) |  |  |  | 715 (83.2) | 144 (16.8) |  |  |
| Chronic lung disease |  |  |  |  |  |  |  |  |  |  |  |  |  |  |
| Yes | 36 (87.8) | 5 (12.2) | a | 0.045 |  | 40 (97.6) | 1 (2.4) | a | 0.18 |  | 30 (73.2) | 11 (26.8) | 2.18 | 0.140 |
| No | 797 (95.4) | 38 (4.6) |  |  |  | 832 (99.6) | 3 (0.4) |  |  |  | 696 (83.4) | 139 (16.6) |  |  |
| Hypertension |  |  |  |  |  |  |  |  |  |  |  |  |  |  |
| Yes | 80 (82.5) | 17 (17.5) | a | <0.001 |  | 96 (99.0) | 1 (1.0) | a | 0.38 |  | 62 (63.9) | 35 (36.1) | 26.15 | <0.001 |
| No | 753 (96.7) | 26 (3.3) |  |  |  | 776 (99.6) | 3 (0.4) |  |  |  | 664 (85.2) | 115 (14.8) |  |  |
| Cardiovascular disease |  |  |  |  |  |  |  |  |  |  |  |  |  |  |
| Yes | 23 (74.2) | 8 (25.8) | a | <0.001 |  | 31 (100) | 0 (0) | a | 1.00 |  | 14 (45.2) | 17 (54.8) | 29.52 | <0.001 |
| No | 810 (95.9) | 35 (4.1) |  |  |  | 841 (99.5) | 4 (0.5) |  |  |  | 712 (84.3) | 133 (15.7) |  |  |

a Fisher's exact test.

b Non-severe includes mild and moderate cases.

1. Li Y, Wei B, Zhang Y. Analysis of the hospitalization expense and structure of 105 COVID-19 patients in Shenzhen. Chinese Hospital Management. (2020) 40(3): p. 42-44.

2. Sun C. (2020). The mean medical costs of severe COVID-19 cases was more than 150 thousand, and all costs were claimed by governments, The Central Commission for Discipline Inspection. https://www.ccdi.gov.cn/yaowen/202004/t20200411_215163.html;2020. [Accessed November13,2020].

3. Hindustantimes. (2020). What does it cost to treat a coronavirus patient? Here’s a break up. https://www.hindustantimes.com/india-news/what-does-it-cost-to-treat-a-coronavirus-patient-here-s-a-break-up/story-qwOzwoaJKj39AAxb2U58oO.html. [Accessed November13,2020].

4. CNBC. (2020). Here’s what you need to know when it comes to paying for coronavirus treatment. https://www.cnbc.com/2020/04/09/heres-what-you-need-to-know-about-coronavirus-treatment-costs.html. [Accessed November13,2020].

5. Time. (2020). Total cost of her COVID-19 treatment: $34,927.43. 2020. https://time.com/5806312/coronavirus-treatment-cost/. [Accessed November13,2020].

6. Zhihu. (2020). Free treatment of new coronavirus for foreigners in Indonesi, 100-200 million Dunn per person. https://zhuanlan.zhihu.com/p/133377631. [Accessed November13,2020].

7. Nation D. (2020). Amoth: Treating coronavirus costs Sh300,000. 2020. https://nation.africa/kenya/news/treating-coronavirus-costs-sh300-000-1475490. [Accessed November13,2020].

8. Healthworld E. (2020). South Africa government, private hospitals agree deal on COVID-19 patients. https://health.economictimes.indiatimes.com/news/hospitals/south-africa-government-private-hospitals-agree-deal-on-covid-19-patients/76251842; 2020. [Accessed November13,2020].

9. Xinhuanet. (2020).Treatment cost of new coronavirus in South Korea: RMB 20000-400000. http://www.xinhuanet.com/world/2020-05/08/c_1210607824.htm. [Accessed November13,2020].

10. Sputniknews. (2020). The treatment cost for severe new coronavirus infection in Russia is about 200000 rubles. http://sptnkne.ws/Duxg. [Accessed November13,2020].

11. PRTIMES. (2020). In the second wave of new coronary pneumonia, 14% of hospitals have taken measures, but there are still obstacles in the supply of masks (GHC survey). https://prtimes.jp/main/html/rd/p/000000007.000046782.html. [Accessed November13,2020].

12. KFF. (2020). Estimated cost of treating the uninsured hospitalized with COVID-19. https://www.kff.org/uninsured/issue-brief/estimated-cost-of-treating-the-uninsured-hospitalized-with-covid-19/. [Accessed November13,2020].

13. CRIOline. (2020). Germany will assume novel coronavirus pneumonia in France, Italy and other European Union countries. http://news.cri.cn/20200420/197ec5a3-d3f6-f1c7-6560-8b59ef0b474d.html. [Accessed November13,2020]

14. CNBC. (2020). Gilead’s coronavirus treatment remdesivir to cost $3,120 per U.S. patient with private insurance. https://www.cnbc.com/2020/06/29/gileads-coronavirus-treatment-remdesivir-to-cost-3120-for-us-insured-patients.html. [Accessed November13,2020].

15. Daily Peoples. (2020). American congresswoman accounts on the spot, forcing the CDC to promise free testing. https://baijiahao.baidu.com/s?id=1661105431846903744. [Accessed November13,2020].

16. Healthline. (2020). What is the cost of getting tested and treated for the coronavirus?.https://www.healthline.com/health-news/if-you-get-the-coronavirus-what-will-it-cost-you#What-about-other-medical-costs? . [Accessed November13,2020].
